# A Non-Intrusive Method for Monitoring the Degradation of MOSFETs

**DOI:** 10.3390/s140101132

**Published:** 2014-01-10

**Authors:** Li-Feng Wu, Yu Zheng, Yong Guan, Guo-Hui Wang, Xiao-Juan Li

**Affiliations:** 1 College of Information Engineering, Capital Normal University, Beijing 100048, China; E-Mails: zhengyu.hello@163.com (Y.Z.); gxy169@sina.com (Y.G.); wgh_boy@126.com (G.-H.W.); lixj@mail.cnu.edu.cn (X.-J.L.); 2 Beijing Engineering Research Center of High Reliable Embedded System, Capital Normal University, Beijing 100048, China; 3 Beijing Key Laboratory of Electronic System Reliable Technology, Capital Normal University, Beijing 100048, China

**Keywords:** non-intrusive, degradation, Volterra series, MOSFET

## Abstract

Highly reliable embedded systems have been widely applied in the fields of aerospace, nuclear power, high-speed rail, *etc.*, which are related to security and economic development. The reliability of the power supply directly influences the security of the embedded system, and has been the research focus of numerous electronic information and energy studies. The degradation of power modules occupies a dominant position among the key factors affecting the power supply reliability. How to dynamically determine the degradation state and forecast the remaining useful life of working power modules is critical. Therefore, an online non-intrusive method of obtaining the degradation state of MOSFETs based on the Volterra series is proposed. It uses the self-driving signal of MOSFETs as a non-intrusive incentive, and extracts the degradation characteristics of MOSFETs by the frequency-domain kernel of the Volterra series. Experimental results show that the identification achieved by the method agrees well with the theoretical analysis.

## 1. Introduction

Switched-mode power supplies (SMPSs) are becoming increasingly common in highly reliable embedded systems, such as aerospace, nuclear power, high-speed rail, *etc.* [1]. The failure of SMPS is directly caused by faults occurring in the system. Moreover, according to the statistics, approximately 34% of electronic system failures result from SMPS failures [2]. The electrolytic capacitors and power semiconductors (MOSFET) are the most likely to fail in SMPS.

Many technologies are available to detect online the failure of electrolytic capacitors. In the case of MOSFETs, most approaches have been proposed to detect them post-fault, including short-circuit and open-circuit faults. Previous work on MOSFETs has focused primarily on three aspects. The first is the reliability design of these components [3]. The second is on predicting the remaining useful life of MOSFETs using off-line accelerated aging tests [4]. An accelerated aging system for the prognostics of discrete power semiconductor devices was built in [5]. Based on accelerated aging with an electrical overstress on the MOSFETs, predictions by gate-source voltage are made in [6]. In [7], collector-emitter voltage is identified as a health indicator. In [8], the maximum peak current of the collector-emitter ringing at the turn off transient is identified as the degradation variable. The third aspect of focus is on the development of degradation models. Degradation models are set up according to the function of the usage time based on accelerated life tests [9]. For example, gate structure degradation modeling of discrete power MOSFETs exposed to ion impurities was presented in [10]. Above all, traditional studies on the degradation of MOSFETs have focused on analyzing non-real time data. Predictions of the remaining useful life of MOSFETs have been based on off-line, statistical analyses.

In recent years, Prognostics and Health Management (PHM) has resulted in a broad range of applications. Many works pay attention to on-line monitoring technology. Papers have proposed algorithms to extract features to monitor MOSFETs and IGBTs in real-time, but the features are difficult to measure accurately. Paper [11] presents a real-time method by capturing the changes of ringing signals to diagnose the health state of IGBTs, but no takes account of the nonlinear features of electronic components.

In this paper, an online non-intrusive method of obtaining the degradation state of MOSFETs based on Volterra series is proposed. We first use the self-driving signals of MOSFETs as a non-intrusive incentive, and extract the degradation characteristics of MOSFETs using the frequency-domain kernel of the Volterra series. According to the relationship between health state and kernel, the state of MOSFETs can be given in real-time and the remaining useful life can be predicted, which can help avoid the inconveniences of fatal accidents, so we have time to deal with the occurrence of faults to realize prognosis and manage the health of electronics. This introductory section is followed by Section 2, which begins by addressing the basic features of MOSFETs and failure mechanisms. Then, based on the Volterra series a transform method is proposed. In Section 3, the experimental procedure is described and how to deal with the data process is discussed. In Section 4, the results of the study are discussed. In Section 5, the conclusions are given.

## 2. Method Based on the Volterra Series

**Figure 1.**
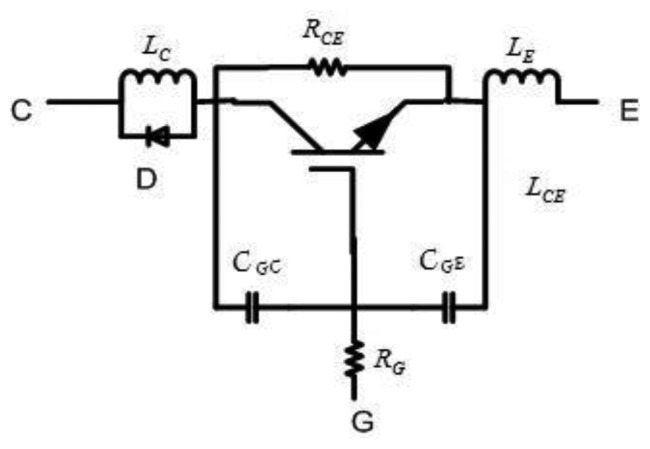
The actual equivalent circuit of a MOSFET.

MOSFETs are widely used in electric systems as ideal on-off switches, but some fail to take into account their actual equivalent circuit, which is shown in Figure 1. It has energy storage element distributed parameters such as inductor, capacitor, and resistance (*i.e.*, *C_GE_*, *C_GE_*, *L_C_*, *L_E_*, *R_G_*, *etc.*), which have inherent nonlinear characteristics.

**Figure 2.**
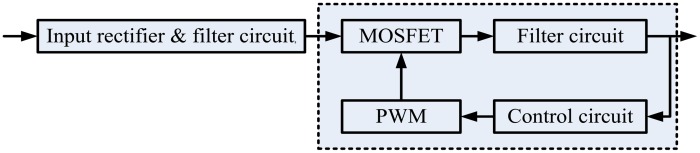
The SMPS topology structure.

From the topology structure of SMPS, which is represented in Figure 2, the MOSFET driven by PWM is a complex memory function and nonlinear dynamic system. Due to aging, environmental impacts and dynamic loading, the MOSFET will degrade over time. The failure mechanisms of MOSFETs include intrinsic mechanisms and transistor packaging. Dielectric breakdowns, electro- migration, bond-wire lift, die-solder degradation and contacts can cause the failure of MOSFETs. All the failures lead to changes in their inherent characteristics. However, the distributed parameters are difficult to measure directly. All the above parameters changes can be equivalent to the change of the internal R, L, C. According to the system theory, given the constant input signal, the output signal will change when the system itself changes. When internal parameters are changing, the response signal will change too.

Based on the nonlinear theory, the output signal y(t) is expressed by:

(1) $$\text{y}(\text{t})=\sum_{n=0}^{\infty }y_{n}(t)$$

in which

(2) $$y_{n}(t)=\int_{-\infty }^{\infty }\cdots \int_{-\infty }^{\infty }h_{n}\;(\tau_{1},\tau_{2},\cdots ,\tau_{n})\prod_{i=1}^{n}u(t-\tau_{i})d\tau_{i}$$

where *y_n_*(*t*) is time domain response, *u*(*t* − *τ_i_*) is input signal, and *h_n_*(*τ*_1_, *τ*_2_, ⋯, *τ_n_*) are the Volterra kernel. The *h_n_*(*τ*_1_, *τ*_2_, ⋯, *τ_n_*) can be expressed by a multidimensional Fourier transform as follows:

(3) $$H_{n}(\omega_{1},\omega_{2},\cdots ,\omega_{n})=\int_{-\infty }^{\infty }\cdots \int_{-\infty }^{\infty }h_{n}(\tau_{1},\tau_{2},\cdots ,\tau_{n})\text{exp}\lceil -j(\omega_{1},\omega_{2},\cdots ,\omega_{n})\rceil d\tau_{1}d\tau_{2}\cdots d\tau_{n}$$

where *H_n_*(*ω*_1_, *ω*_2_, ⋯ *ω_n_*) is generalized frequency response function (GFRF).

Given the excitation signal is a square wave signal, it can be expressed in the following form:

(4) $$\text{u}(t)=\sum_{i=1}^{K}|A_{i}|\cos(\omega_{i}t+\theta_{i})=\sum_{\begin{array}{c} i=-K\\ i\neq 0 \end{array}}^{K}\frac{A_{i}}{2}\exp(j\omega_{i}t)$$

Considering the actual application, we only use the first three frequencies to replace the square wave signal. So, the u(t) can be given as follows:

(5) $$\text{u}(\text{t})≅|A_{1}|\cos(\omega_{1}t+\theta_{1})+|A_{2}|\cos(\omega_{2}t+\theta_{2})+|A_{3}|\cos(\omega_{3}t+\theta_{3})$$

According to Equations (1), (2) and (5), it is clear that the output frequency depends on the inherent characteristics. From the theory analysis, we can know that when the health of a MOSFET changes, the output frequency will change accordingly, so the degradation of the MOSFET can be mapped to the changing amplitudes of different frequency. Here, we extract the amplitude of the third order nuclear frequency as the feature parameters for the degradation of the MOSFET.

According to the experiments, the relationship between life state (LS) and degradation state can be expressed as follows:

(6) $$\{\begin{array}{c} \text{LS}=a_{k}\overset{i=n,j=m}{\underset{i=1,j=1}{\text{∑}}}\frac{B_{i,j}}{A_{i,j}}\\ k=m\\ \sum a_{k}=1 \end{array}$$

where *Ai,j* is the normal MOSFET amplitude of i-order nuclear and j-frequency. *B_i,j_* is the degradation of the MOSFET amplitude of i-order nuclear and j-frequency. *a_k_* is the coefficient, which can be determined by tests, so we can know the life state according to Equation (6).

## 3. Experiments and Feature Data Extraction

**Figure 3.**
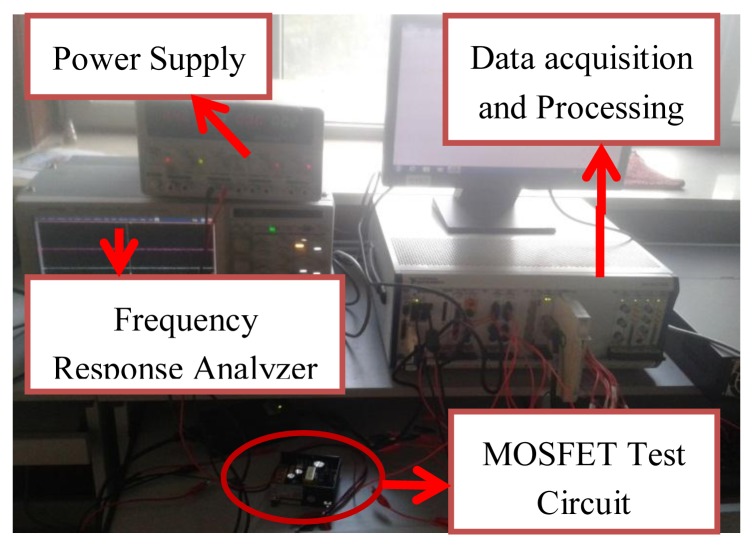
Experimental setup.

The performance of the presented monitoring of MOSFET degradation was verified by physical experiments. The test system is shown in Figure 3, and includes: (1) the SMPS, whose MOSFET is considered pluggable, making it easy to replace a MOSFET with another of the same type but with varying degrees of degradation; (2) Temperature control chamber. MOSFETs undergo accelerated degradation using thermal overstress; (3) High precision voltage sensor, where the input and output signals are transformed to send to the data acquisition card; (4) High speed, high precision data acquisition card, collecting excitation signals and output signals on a PC.

**Figure 4.**
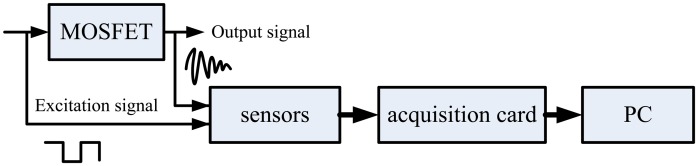
Diagram of the data acquisition process.

**Figure 5.**
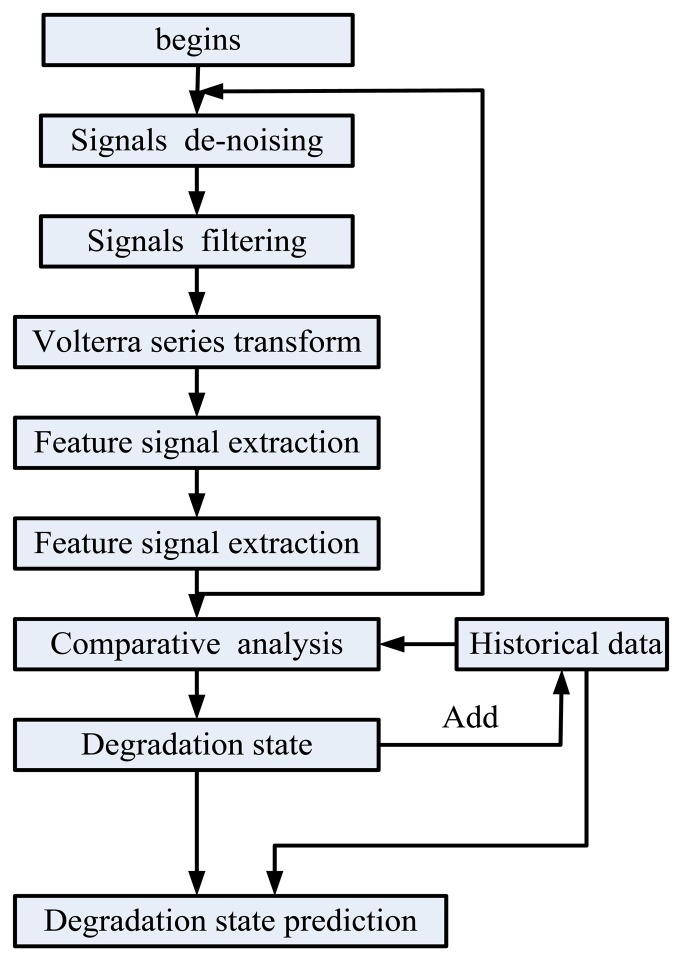
Program flow chart of degradation state judgment and prediction for MOSFET.

The data acquisition and processing is shown in Figure 4. First, we obtain the excitation signal and output signal suing high precision voltage sensors. Then the signals are acquired by a NI acquisition card and sent to the PC. A program is developed to deal with data based on the Labview software. The program flow chart is shown in Figure 5.

In order to verify the method, we use ten MOSFETs, which have different degrees of degradation. Here, we take advantage of the temperature change to obtain the MOSFETs with different degradation. The MOSFETs are put into the circuit, respectively. First, we denoise the transient response to a square control signal at the gate, and then use FIR method to filter the signals. Based on the Volterra series transform, we extract the feature signals of the degradation, that is, the first three order frequency nuclear signals. By comparison of the current state and historical data, we can obtain information about two aspects. On the one hand, we can know the MOSFET degradation. On the other hand, we can obtain the nonlinear relationship between the remaining useful life and time by the least squares method, so future degradation trends can be predicted.

## 4. Results and Discussion

**Figure 6.**
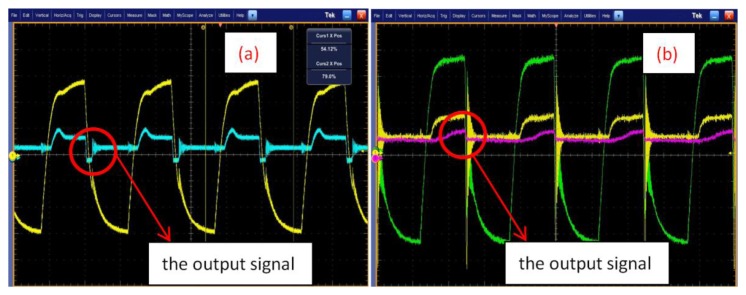
The the output signal. (**a**) MOSFET is normal; (**b**) MOSFET is degraded.

**Figure 7.**
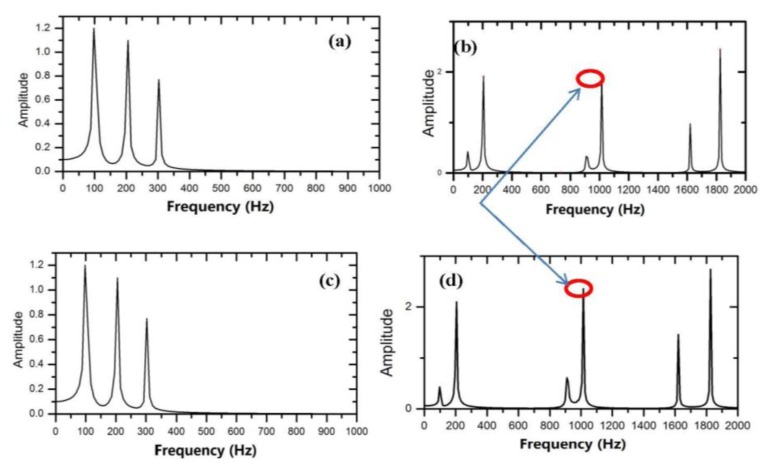
The Volterra series transform for the output signal. (**a**,**b**) First-order and second-order nuclear output spectra, respectively, when the MOSFET is normal; (**c**,**d**) First-order and second-order nuclear output spectra, respectively, when the MOSFET is degraded.

Figure 6 depicts the output signal. Figure 6a,b is the output signals, when MOSFET is normal and degraded, respectively. Figure 7 depicts the Volterra series transform for the output signal. Figure 7a,b is the first-order and second-order nuclear output spectrum, respectively, when the MOSFET is normal.

We take advantage of some degradation data set as a training sample to identify the parameters of Equation (6) and use the other degradation data to test it. Table 1 represents the compared results of the actual and predicted values. As shown in Table 1, the average error between the actual and the estimated results in this study are 4%–6% from the seven groups of data.

## 5. Conclusions

In this paper, a non-intrusive online method for obtaining the degradation state of MOSFETs is developed based on the Volterra series. We use the self-driving signal of MOSFETs as non-intrusive incentives, and extract the degradation characteristics of MOSFETs with the frequency-domain kernel of the Volterra series. Simulation and experimental results on the state of degradation of MOSFETs have been compared and analyzed. A good correlation between prediction and actual results has been demonstrated. Compared to the previous methods, the proposed approach has several advantages to avoid the inconveniences due to MOSFETs' sudden failure and enormous repair costs.

## Figures and Tables

**Table 1.** Comparison between prediction and actual results for the degradation state of a MOSFET.

**Group**	**Actual Life State**	**Predicted Life State**
1	100%	100%
2	90%	85%
3	80%	83%
4	70%	75%
5	60%	64%
6	50%	56%
7	40%	46%
